# Post-VX exposure treatment of rats with engineered phosphotriesterases

**DOI:** 10.1007/s00204-021-03199-6

**Published:** 2021-12-28

**Authors:** Lisa Stigler, Anja Köhler, Marianne Koller, Laura Job, Benjamin Escher, Heidrun Potschka, Horst Thiermann, Arne Skerra, Franz Worek, Timo Wille

**Affiliations:** 1 Bundeswehr Institute of Pharmacology and Toxicology, Neuherbergstraße 11, 80937 Munich, Germany; 2 Chair of Biological Chemistry, Technical University of Munich, Emil-Erlenmeyer-Forum 5, 85354 Freising, Germany; 3 Institute of Pharmacology, Toxicology and Pharmacy, Ludwig-Maximilians-University Munich, Königinstraße 16, 80539 Munich, Germany

**Keywords:** Acetylcholinesterase, Catalytic bioscavenger, Detoxification, Enzyme engineering, In vivo, Organophosphate, PASylation, Phosphotriesterase, Therapy, VX

## Abstract

The biologically stable and highly toxic organophosphorus nerve agent (OP) VX poses a major health threat. Standard medical therapy, consisting of reactivators and competitive muscarinic receptor antagonists, is insufficient. Recently, two engineered mutants of the *Brevundimonas diminuta* phosphotriesterase (PTE) with enhanced catalytic efficiency (*k*_cat_/*K*_M_ = 21 to 38 × 10^6^ M^−1^ min^−1^) towards VX and a preferential hydrolysis of the more toxic P(−) enantiomer were described: PTE-C23(R152E)-PAS(100)-10-2-C3(I106A/C59V/C227V/E71K)-PAS(200) (PTE-2), a single-chain bispecific enzyme with a PAS linker and tag having enlarged substrate spectrum, and 10-2-C3(C59V/C227V)-PAS(200) (PTE-3), a stabilized homodimeric enzyme with a double PASylation tag (PAS-tag) to reduce plasma clearance. To assess in vivo efficacy, these engineered enzymes were tested in an anesthetized rat model post-VX exposure (~ 2LD_50_) in comparison with the recombinant wild-type PTE (PTE-1), dosed at 1.0 mg kg^−1^ i.v.: PTE-2 dosed at 1.3 mg kg^−1^ i.v. (PTE-2.1) and 2.6 mg kg^−1^ i.v. (PTE-2.2) and PTE-3 at 1.4 mg kg^−1^ i.v. Injection of the mutants PTE-2.2 and PTE-3, 5 min after s.c. VX exposure, ensured survival and prevented severe signs of a cholinergic crisis. Inhibition of erythrocyte acetylcholinesterase (AChE) could not be prevented. However, medulla oblongata and diaphragm AChE activity was partially preserved. All animals treated with the wild-type enzyme, PTE-1, showed severe cholinergic signs and died during the observation period of 180 min. PTE-2.1 resulted in the survival of all animals, yet accompanied by severe signs of OP poisoning. This study demonstrates for the first time efficient detoxification in vivo achieved with low doses of heterodimeric PTE-2 as well as PTE-3 and indicates the suitability of these engineered enzymes for the development of highly effective catalytic scavengers directed against VX.

## Introduction

Despite ban by the Chemical Weapons Convention, highly toxic organophosphorus nerve agents (OP) were repeatedly used in military conflicts, terrorist attacks or assassination attempts. The use of sarin in the Syrian civil war, the supposed VX poisoning of Kim Jong Nam 2017 as well as the homicidal attacks of Sergei Skripal 2018 and of Alexei Navalny 2020 with novichok underline the persistent threat of these vicious compounds (Berlinger 2017; Dewan and Alkashli 2017; Halasz et al. 2020; John et al. 2018; Steindl et al. 2021; Vale et al. 2018).

The high toxicity of OP results from covalent binding to and subsequent inhibition of the pivotal enzyme acetylcholinesterase (AChE). This leads to a synaptic overflow of acetylcholine (ACh), followed by overstimulation and desensitization of muscarinic and nicotinic cholinergic receptors causing a broad spectrum of clinical signs including miosis, salivation and cardiac arrhythmia (Aldridge and Davison 1953; Aldridge and Reiner 1972; Holmstedt 1959). Ultimately, central and peripheral respiratory depression may lead to death by asphyxia (Thiermann et al. 2013; Yanagisawa et al. 2006).

For the past 60 years the standard therapy of OP poisoning has been a combination of the competitive muscarinic receptor antagonist atropine and an oxime as AChE reactivator (Cannard 2006; Thiermann et al. 2013). However, multiple in vitro and in vivo studies have shown that this treatment has limited effectiveness against different OP and cannot prevent cholinergic signs (Thiermann et al. 2013; Worek and Thiermann 2013). This triggered extensive research on enzyme-based scavengers to detoxify OP via hydrolysis to less toxic products in the blood compartment, thus preventing the distribution into target tissues and reducing toxicity (Masson and Nachon 2017; Worek et al. 2016a).

Phosphotriesterase (PTE) from *Brevundimonas diminuta* has emerged as a promising candidate for a catalytic bioscavenger (Masson and Rochu 2009; Worek et al. 2016a). Several research groups described PTE variants with improved catalytic activity, broadened substrate spectrum and stereopreference towards the more toxic P(−) nerve agent enantiomer (Bigley et al. 2013, 2015; Cherny et al. 2013; Goldsmith et al. 2017; Worek et al. 2016a). Hereby, a main focus was to improve the detoxification of highly toxic and biologically stable V-agents such as VX (Benschop and de Jong 1988; Masson and Nachon 2017; Reiter et al. 2015).

In fact, post-exposure therapy of VX poisoned guinea pigs with the engineered PTE mutant C23 (*k*_cat_/*K*_M_ = 5 × 10^6^ M^−1^ min^−1^) ensured survival and prevented systemic cholinergic toxicity (Worek et al. 2014), indicating that PTE variants may have the potential for use as pre- and post-exposure prophylaxis as well as post-exposure treatment (Worek et al. 2016b). Another improved mutant, the PTE variant C23AL detoxified VX with *k*_cat_/*K*_M_ = 12 × 10^6^ M^−1^ min^−1^, but in vivo data showed slower kinetics than the expected breakdown of VX extrapolated from in vitro data (Wille et al. 2016).

Theoretical calculations by Ashani et al. (2016) and Worek et al. (2016a) suggest a catalytic enzyme activity of > 50 × 10^6^ M^−1^ min^−1^ as a prerequisite for a practically applicable enzyme dose of ≤ 1 mg kg^−1^ body weight to achieve a VX degradation half-life < 5 s for sufficient detoxification. Therefore, new mutants were recently engineered based on previously published variants C23 (Cherny et al. 2013), 10-2-C3 and 10-2-C3-I106A (Goldsmith et al. 2017).

Novel variants were generated to improve catalytic efficacy and stability, e.g. by replacing two unpaired Cys residues by the more inert amino acid Val (Job et al. 2020) or by creating a highly effective PTE heterodimer with broadened substrate spectrum (Escher et al. 2020). These two promising mutants were optimized with regard to extended plasma half-life with an additional C-terminal PAS-tag (Schlapschy et al. 2013) and showed efficient detoxification in vitro towards a broad substrate spectrum of structurally diverse nerve agents (Köhler et al. 2021).

Consequently, we have now set out to test the efficacy of the two novel PTE mutants, PTE-2 and PTE-3 (Table 1), in comparison with wild-type PTE (PTE-1) in a post-exposure therapy model of VX exposed rats.

**Table 1:** Overview of PTE variants tested in vivo

	Variant	Molecular mass (Da)
PTE-1	Wild-type PTE^a, b^	37,617.9^e^
PTE-2	PTE-C23(R152E)-PAS(100)-10-2-C3(I106A/C59V/C227V/E71K)-PAS(200)^c, d^	98,502.3
PTE-3	10-2-C3(C59V/C227V)-PAS(200)^b, c^	54,075.9^e^

^a^Caldwell and Raushel (1991)

^b^Job et al. (2020)

^c^Köhler et al. (2021)

^d^Escher et al. (2020)

^e^These enzymes are non-covalent hetero-dimers, only the mass of one subunit is shown

## Materials and methods

### Chemicals

The OP VX (*O*-ethyl S-(diisopropylaminoethyl)phosphonothioate; > 98% by ^1^H and ^31^P NMR) and VR (*O*-isobutyl S-(diethylaminoethyl)phosphonothioate; > 98% by ^1^H and ^31^P NMR) were made available by the German Ministry of Defence. VX and VR stock solutions (1% v/v in acetonitrile) were stored at room temperature. For each in vivo test, a fresh VX working solution (36 µg mL^−1^) was prepared in saline and put on ice until use. For the stereoselective analytics of VX in rat blood samples a VR working solution (20 ng mL^−1^) was prepared in acetonitrile as internal standard.

Tris[hydroxymethyl]-aminomethane (TRIS), Triton X-100, 5,5'-dithiobis(2-nitrobenzoic acid) (DTNB), acetylthiocholine iodide (ATCh), and ethopropazine were purchased from Sigma–Aldrich (Taufkirchen, Germany). Human erythrocyte acetylcholinesterase (AChE) was prepared from heparinized whole blood according to Bierwisch et al. (2014), Dodge et al. (1963), Worek et al. (2002). All other chemicals were purchased from Merck (Darmstadt, Germany).

### Protein expression and purification

PTE-1, PTE-2, and PTE-3 (Table 1) were expressed in *Escherichia coli* using previously described plasmids according to published procedures by Escher et al. (2020), Job et al. (2020) and Köhler et al. (2021). For production of PTE-1, *E. coli* BL21 was transformed with the expression plasmid encoding wild-type-PTE, whose synthetic gene was inserted between the *Kas*I and *Hin*dIII restriction sites on pASK-IBA5( +) (IBA, Göttingen, Germany), including an N-terminal *Strep*-tag II (Schmidt and Skerra 2007). The bacteria were cultivated at 22 °C in shake flasks with 2 L TB medium (0.017 mol L^−1^ KH_2_PO_4_, 0.072 mol L^−1^ K_2_HPO_4_, 12 g L^−1^ Bacto tryptone, 24 g L^−1^ Bacto yeast extract, 4 ml L^−1^ glycerol) containing ampicillin (100 mg L^−1^) and ZnSO_4_ (0.2 mmol L^−1^). Recombinant gene expression was induced at OD_550_ ≈ 1.8–2.2 by adding anhydrotetracycline (200 µg L^−1^) for up to 15 h. Bacteria were harvested by centrifugation (40 min, 5016 × *g*, 4 °C), resuspended in 3 mL affinity chromatography buffer (100 mmol L^−1^ TRIS/HCl, 150 mmol L^−1^ NaCl, 10 mmol L^−1^ NaHCO_3_, 0.1 mmol L^−1^ ZnSO_4_, pH 8.0) per 1 g wet weight and mechanically disrupted using a high-pressure homogenizer (PandaPLUS 2000; GEA Niro Soavi, Lübeck, Germany). The soluble extract was loaded onto a *Strep*-Tactin column for affinity purification as described (Schmidt and Skerra 2007), followed by dialysis overnight against 20 mmol L^−1^ Hepes/NaOH, 20 mmol L^−1^ NaCl, 10 µmol L^−1^ ZnSO_4_ pH 7.2 for PTE-1 and against 20 mmol L^−1^ Bis–Tris/HCl, 20 mmol L^−1^ NaCl, 10 µmol L^−1^ ZnSO_4_ pH 6.0 for PTE-3. To remove endotoxin, the homodimeric PTE variants were loaded onto a 6 mL Resource Q anion exchange column (ResQ; Cytiva, Freiburg, Germany) equilibrated with the dialysis buffer. The protein was collected from the flow through and wash fractions. To remove the oligomers of the PTE-2, an anion-exchange chromatography (AEX) was applied using a 6 mL ResQ column (Cytiva, Freiburg, Germany) equilibrated with 20 mmol L^−1^ Hepes/NaOH, 20 mmol L^−1^ NaCl, 10 µmol L^−1^ ZnSO_4_ at pH 7. The different species were separated by a linear concentration gradient from 20 to 250 mmol L^−1^ NaCl in running buffer over 20 column volumes. All PTEs were subjected to size-exclusion chromatography (SEC) on a 120 mL HiLoad Superdex 200 16/60 prep grade column or 320 ml HiLoad Superdex 200 26/60 prep grade column (GE Healthcare, Freiburg, Germany) for final purification and buffer exchange using 50 mmol L^−1^ TRIS/HCl, 100 mmol L^−1^ NaCl, 10 µmol L^−1^ ZnSO_4_ pH 8.0 as running buffer. Endotoxin contents were determined using an Endosafe-PTS system (Charles River Laboratories, Wilmington, MA, USA) and were below 15 EU mg^−1^ for all proteins. Protein concentrations were quantified using an Ultrospec 2100 pro UV–Vis spectrophotometer (Cytiva, Freiburg, Germany) with a molar absorption coefficient at 280 nm calculated according to Wilkins et al. (1998). Protein purity was determined using SDS/PAGE (Coomassie-stained sodium dodecyl sulfate polyacrylamide gel electrophoresis) using the buffer system of Fling and Gregerson (1986) to be > 99% for all enzyme variants.

### Activity measurement of PTE enzymes

The organophosphorus hydrolysis (OPH) rate of VX by the PTE mutant 1 was quantified in vitro as described by Goldsmith et al. (2016), Job et al. (2020), and Köhler et al. (2021). PTE-1 was incubated at 37 °C with VX in 50 mmol L^−1^ TRIS/HCl, 50 mmol L^−1^ NaCl, pH 8.0 (final concentrations in a total volume of 600 µL: PTE-1 9.8 × 10^–6^ mol L^−1^ and VX 3.3 × 10^–6^ mol L^−1^). At defined time points (1, 7, 14, 20, 30, 45, 60, 90, and 120 min), 50 µL aliquots of this solution were added to an assay mixture (total volume; 3.15 mL) containing acetylthiocholine (ATCh; 0.45 mmol L^−1^) and Ellman’s reagent DTNB (0.3 mmol L^−1^) in 0.1 mol L^−1^ phosphate buffer (pH 7.4). To initiate the chromogenic reaction, 10 µL AChE (200 mE/min in phosphate buffer) was added and inhibition curves were recorded at 412 nm at 37 °C for 5 min using a UV-2600 photometer (Shimadzu, Kyoto, Japan). Pseudo-first order rate constants for AChE activity *k*_AChE_(*t*_OPH_) and the OPH constant *k*_OPH_ were obtained and calculated as described by Job et al. (2020) and Köhler et al. (2021). *k*_cat_/*K*_M_ of PTE-2 and PTE-3 were taken from Köhler et al. (2021).

### Animals

Male Wistar rats (approximately 250–370 g; corresponding to an age range of 7–9 weeks) were purchased from Charles River (Sulzfeld, Germany). In total, *n* = 65 animals were used, five died during preparation. Prior to experiments, the animals were housed at least 7 days for acclimatization under controlled conditions (12 h light/dark cycle [light from 7:00 a.m. to 7:00 p.m.], 20–24 °C, 45–65% humidity) with standard diet (Ssniff Spezialdiäten, Soest, Germany) and tap water ad libitum in eurostandard Makrolon cages type IV with heightened lids (Tecniplast, Hohenpeißenberg, Germany). For enrichment a house (Zoonlab, Castrop-Rauxel, Germany), embedding (Rettenmeier&Söhne, Rosenberg, Germany), nestlets and wood wool (both from Zoonlab) were provided and replaced once a week. Every attempt was made to minimize discomfort and pain and to reduce the number of animals used in the study.

All experiments were in conformity with the German Animal Welfare Act of 18th May 2006 (BGBI. I S. 1206, 1313), the European Parliament and Council Directive of 22nd September 2010 (2010/63/EU) and carried out according to the Basel declaration as well as the 3R concept (replace, reduce, refine). The study was ethically approved by the institutional animal protection committee (Ref.-No. 42-34-30-40/G03-19).

### Experimental procedure for PTE plasma concentration profile determination

**Fig. 1:**
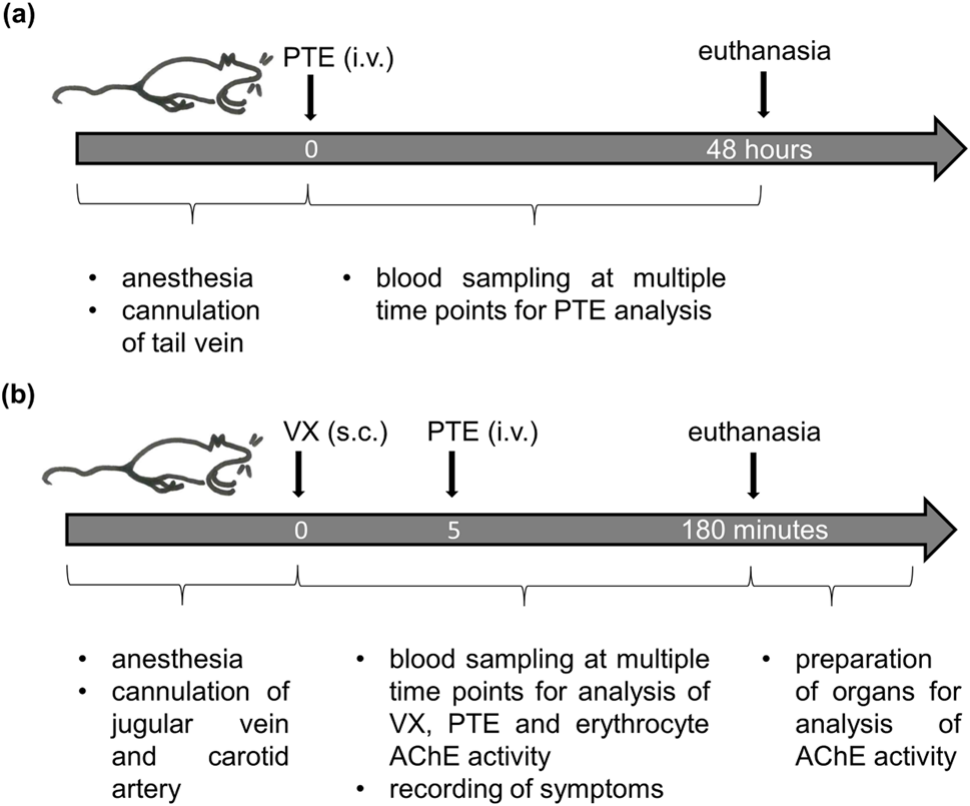
Time lines for the determination of the PTE plasma profile (**a**) and the therapeutic efficacy study (**b**)

For PTE administration and blood sampling as shown in the time line (Fig. 1a), rats were repetitively anesthetized with short-time isoflurane inhalation (IsoFlo 100% w/w, Abbott Laboratories, Burgdorf, Germany; oxygen as carrier gas), 5% for induction of anesthesia and 2% for maintenance. In advance to vascular cannulation, local anesthetic creme (25 mg g^−1^ + 25 mg g^−1^ Prilocaine–Lidocaine; Pierre Fabre, Freiburg, Germany) was applied. To ensure an adequate depth of anesthesia, reflexes were checked regularly. Defined PTE doses were injected into the respective lateral tail vein using a peripheral intravenous inserted catheter (24 Gauge i.v. indwelling cannula, 0.7 × 19 mm; B. Braun, Melsungen, Germany) and flushed with saline before removal. Subsequently, blood samples were taken at various time points from the lateral tail vein over a period of 48 h (10 min, 20 min, 30 min, 60 min, 120 min, 240 min, 480 min, 960 min, 1440 min and 2880 min) with a maximum of five samples per animal. Sterile hypodermic needles were used to prick the vein and the blood samples were collected in heparinized Minivettes (POCT, Sarstedt, Nürmbrecht, Germany).

### Experimental groups for PTE plasma concentration profiles

The animals (*n* = 24) were divided into three groups by simple randomization. PTE-1 was administered at a dose of 1.0 mg kg^−1^, the doses of PTE-2 and PTE-3 were adjusted according to the molecular weight to achieve equal stoichiometries (note that PTE-2 is a single-chain heterodimer with two distinct active sites) (Table 1).

- PTE-1 (1.0 mg kg^−1^ i.v.; *n*=8).
- PTE-2 (1.3 mg kg^−1^ i.v.; *n*=8).
- PTE-3 (1.4 mg kg^−1^ i.v.; *n*=8).

### Experimental procedure for the therapeutic efficacy study

Rats were anesthetized by i.m. injection of a mixture of medetomidine (0.15 mg kg^−1^; Zoetis, Berlin, Germany), midazolam (0.4 mg kg^−1^; Ratiopharm, Ulm, Germany) and fentanyl (0.1 mg kg^−1^; Albrecht, Aulendorf, Germany) to reduce suffering from VX challenge. Reflexes were checked for depth of anesthesia throughout the whole experiment and anesthesia re-dosing was applied as required with 1/4–1/3 of the initial dose. Next, the animals were placed on a heatable operating table in supine position, a rectal thermistor was inserted and an electrocardiogram and a pulse oximeter were attached.

Eyes were covered with panthenol eye cream (Jenapharm Mibe, Brehna, Germany) to protect from dehydration. After preparation of the left a. carotis (for blood sampling) and the right v. jugularis (for PTE injection), catheters (24 G × ¾ [0.7 × 19 mm] Introcan; B. Braun) were inserted and fixed. The animals were monitored (blood pressure, heart rate, oxygenation, body temperature) throughout the whole experiment. Clinical signs were observed and heparinized blood samples (250 µL replaced by Ringer’s solution) were collected. The first blood sample was taken before and 11 further blood collections were scheduled between 3 and 180 min after subcutaneous injection of 25 µg kg^−1^ (~ 2LD_50_) VX prepared in saline (Misik et al. 2015; Myhrer et al. 2015).

For AChE activity determination, 50 µL whole blood was diluted (1:20) in distilled water, whereas 100 µL whole blood was used for VX analysis. The remaining blood sample was centrifuged and plasma was used for PTE analysis. All samples were snap-frozen in liquid nitrogen and stored at −80 °C until use. Directly after the end of the experiment tissue samples of diaphragm and medulla oblongata were taken and their wet weight determined. For the time line see Fig. 1b.

### Experimental groups for the therapeutic efficacy study

The animals (*n* = 36) were divided into six groups by simple randomization:

- Solvent control group (saline i.v. 5 min after saline s.c.; *n* = 6).
- VX control group (saline i.v. 5 min after 25 µg kg^−1^ VX s.c.; *n* = 6).
- PTE-1 treatment group (1.0 mg kg^−1^ PTE-1 i.v. 5 min after 25 µg kg^−1^ VX s.c.; *n* = 8).
- PTE-2.1 treatment group (1.3 mg kg^−1^ PTE-2 i.v. 5 min after 25 µg kg^−1^ VX s.c.; *n* = 4).
- PTE-2.2 treatment group (2.6 mg kg^−1^ PTE-2 i.v. 5 min after 25 µg kg^−1^ VX s.c.; *n* = 4).
- PTE-3 treatment group (1.4 mg kg^−1^ PTE-3 i.v. 5 min after 25 µg kg^−1^ VX s.c.; *n* = 8).

### PTE analysis

PTE concentrations in plasma samples were determined by means of the enzymatic activity via hydrolysis of paraoxon-ethyl (PXE) as a chromogenic substrate. For calibration curves of each PTE mutant, 2 µL of the purified recombinant enzyme solution was diluted in TZN buffer (50 mmol L^−1^ TRIS/HCl, 10 µmol L^−1^ ZnSO_4,_ 50 mmol L^−1^ NaCl, pH 8.0) and added to 48 µL heparinized plasma from untreated rats up to final PTE concentrations between 1 and 10 µg mL^−1^. Plasma samples from the in vivo study were diluted in TZN buffer (PTE-1 and PTE-3, 1:5; PTE-2, 1:10). After incubation on ice for 2 h, all samples were further diluted (PTE-1, 1:80; PTE-2, 1:20; PTE-3, 1:40) in TZN buffer. Then, 150 µL PXE substrate solution (1 mmol L^−1^ in TZN buffer) was mixed each with 50 µL sample solution in a 96-well plate (cell culture plate, 96-well, PS, flat bottom; Greiner Bio-One, Frickenhausen, Germany). The change in absorbance at 400 nm was immediately recorded using an infinite M200 PRO plate reader (Tecan, Wien, Austria) at 25 °C for 2 min. The time-dependent absorbance of samples from the calibration curve and plasma were calculated in mA min^−1^. Pre-determined calibration curves were used for the calculation of the enzyme level in plasma samples. The corresponding circulation half-life $\left( {{\text{PTE~}}t_{{{1 \mathord{\left/ {\vphantom {1 2}} \right. \kern-\nulldelimiterspace} 2}}} } \right)$ was obtained using a one-compartment model with a mono-exponential decay of the calculated plasma enzyme levels versus the blood collection time points, and the maximal (initial) plasma concentration of PTE variants (*C*_max_) was estimated by retrograde extrapolation of the mono-exponential curve fit using Prism Version 4.03 (GraphPad, San Diego, CA, USA).

### AChE activity assay

Diaphragm and medulla oblongata were removed after the end of the experiment or death of the animal, snap-frozen in liquid nitrogen and stored at −80 °C until the determination of the AChE activity.

Diaphragm was mixed with a tenfold volume of TRIS/HCl buffer (50 mmol L^−1^, pH 7.4), supplemented with EDTA (5 mmol L^−1^) and 1% v/v Triton X-100 followed by homogenization of the mixture with a T 25 digital ULTRA-TURRAX (IKA, Staufen, Germany). Medulla oblongata was mixed with a tenfold volume of phosphate buffer (0.1 mol L^−1^, pH 7.4) containing 1% v/v Triton X-100. This mixture was subsequently homogenized with a glass-Teflon Potter (B. Braun). Afterwards, the homogenate was centrifuged (Microfuge 22; Hettich, Tuttlingen, Germany) at maximum speed for 5 min. The supernatant was used to measure the AChE activity according to a modified Ellman assay (Worek et al. 1999). The total protein concentration was determined with a bicinchoninic acid assay (Protein Quantification Kit; Interchim Uptima, Montlucon, France) using bovine serum albumin as standard (Smith et al. 1985).

For analysis of all in vivo AChE activities polystrol cuvettes were filled with phosphate buffer (0.1 mol L^−1^, pH 7.4), 0.3 mmol L^−1^ DTNB and 0.02 mmol L^−1^ ethopropazine. After adding the sample the assay was started by adding 0.45 mmol L^−1^ ATCh and the absorbance change was recorded at 436 nm at 37 °C (Cary 50; Varian, Darmstadt, Germany). Erythrocyte AChE activity was referenced to the hemoglobin concentration of the respective blood dilution (Worek et al. 1999) and was calculated as mU µmol^−1^ Hb. Diaphragm and brain AChE activity were referenced to the total protein concentration determined by the bicinchoninic acid method (Smith et al. 1985) and presented as mU mg^−1^.

### Analysis of VX in blood samples

VX enantiomers in blood samples were quantified by LC–MS/MS as described before (Reiter et al. 2008, 2011) with slight modifications. 100 µL thawed, heparinized rat whole blood was diluted with 300 µL water and mixed with 37.5 µL 1 mol L^−1^ perchloric acid, followed by addition of 7.5 µL 1 mol L^−1^ potassium acetate, and vortexed. The precipitated proteins were separated by centrifugation for 10 min at 21,290 × *g* at 4 °C. The supernatant was mixed with 50 µL of 0.5% w/v ammonium hydroxide and 5 µL of internal standard solution (20 ng mL^−1^ VR working solution prepared in acetonitrile) and centrifuged again for 3 min at 21,290 × *g* at 4 °C. The sample was applied to a preconditioned (1 mL methanol, 1 mL purified water; Milli-Q, Merck Millipore, Schwalbach, Germany) SPE cartridge (Strata-X PRP, 30 mg, 1 mL; Phenomenex, Aschaffenburg, Germany) on a vacuum manifold. The column bed was rinsed with 1 mL water and carefully dried under maximum vacuum. The sample was eluted with 500 µL acetonitrile and collected in a cup filled with 50 µL purified water as keeper. The organic phase was evaporated in a vacuum rotation concentrator (50 min at room temperature) with 11,360 x *g* and the residue was reconstituted with 100 µL purified water.

The analytical LC–MS/MS system for the quantification of VX enantiomers consisted of a Prominence HPLC system (Shimadzu Deutschland, Duisburg, Germany) and a QTRAP 6500 mass spectrometer (Sciex, Darmstadt, Germany).

Peak separation was achieved with a Chiralpak AGP column (150 × 2.1 mm, 5 µm, VWR, Darmstadt, Germany) at a flow rate of 175 µL min^−1^ using 25 mmol L^−1^ ammonium formate (pH 8.5) in water (eluent A) and 25 mmol L^−1^ ammonium formate (pH 8.5) in 1:1 methanol–water (eluent B) with the following gradients: 90% A (0–3 min), 90–50% A (3–23 min), 50% A (23–26 min), 50–90% A (26–27 min), 90% A (27–30 min). The injection volume was 10 µL, applied at a column temperature of 30 °C.

Two MRM (multiple reaction monitoring) transitions each for VX and VR (internal standard) were selected for detection after positive electrospray ionization at 5.5 kV with a source temperature of 400 °C and by applying the following declustering potentials and collision energies, respectively: *m/z* 268.1 → 128.1 (120, 25), *m/z* 268.1 → 86.0 (146, 32) for VX and *m/z* 268.1 → 100.1 (120, 25), m/z 268.1 → 72.2 (121, 42) for VR.

Gas 1, gas 2, and curtain gas were set at 35, 40, 45 arbitrary units. The entrance potential was set to 5.0 V, cell exit potential was set to 12 V. Dwell time was 750 ms and resolution was “unit” for both quadrupoles.

### Calculation of VX half-life

The theoretical half-life of VX hydrolysis $\left( {{\text{VX~}}t_{{{1 \mathord{\left/ {\vphantom {1 2}} \right. \kern-\nulldelimiterspace} 2}}} } \right)$ was calculated by Eq. (1) according to Worek et al. (2016a) using in vitro* k*_cat_/*K*_M_ and the maximal plasma concentration of PTE variants (*C*_max_):

1 $${\text{VX~}}t_{{{1 \mathord{\left/ {\vphantom {1 2}} \right. \kern-\nulldelimiterspace} 2}}} = \frac{{{\text{ln}}\left( 2 \right)}}{{{\frac{{k_{{\text{cat}}}}}{{K_{{\text{M}}}}}{\text{~}} \times {\text{~}}C_{{{\text{max}}}} }}}$$

### Data analysis

Data are shown as mean ± standard deviation (SD). Data analysis and statistical comparisons were performed using Prism Version 5.04.

For the analysis of the diaphragm and medulla oblongata AChE activity differences between groups, a one-way repeated measure analysis of variance (ANOVA) with Bonferroni post-hoc test multiple comparison test was used. A *p* < 0.05 value was considered to be statistically significant.

## Results

### PTE plasma concentration profile in vivo

**Fig. 2:**
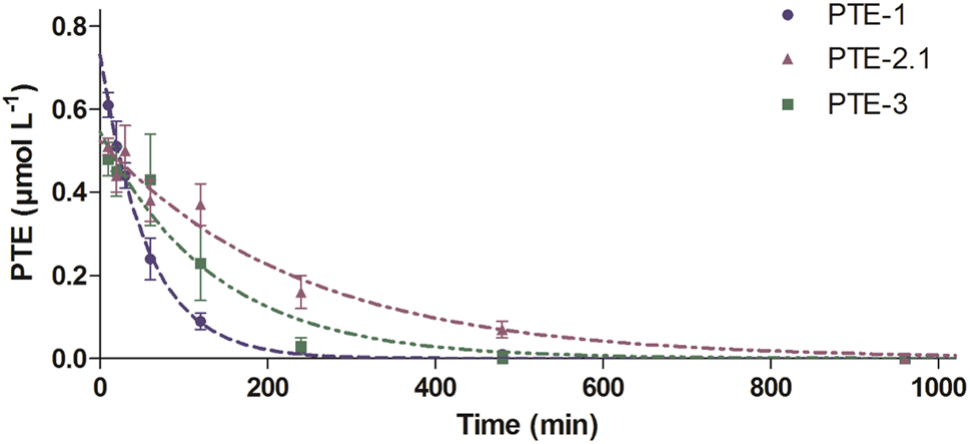
PTE plasma concentration profile in rats over 48 h after injection of PTE-1 (1.0 mg kg^−1^ i.v.), PTE-2 (1.3 mg kg^−1^ i.v.) or PTE-3 (1.4 mg kg^−1^ i.v.)

As a prerequisite to study the protective effect of the newly engineered PTE enzymes, PTE-2 and PTE-3 in comparison to wild-type PTE-1, against VX exposure in rats their pharmacokinetics (PK) after i.v. injection was studied in the same species (Fig. 1a). PTE concentrations were analyzed according to the one compartment model, resulting in plasma half-life values $\left( {{\text{PTE~}}t_{{{1 \mathord{\left/ {\vphantom {1 2}} \right. \kern-\nulldelimiterspace} 2}}} } \right)$ of 39 min, 165 min and 94 min for PTE-1, PTE-2, and PTE-3, respectively (Fig. 2). As expected, the application of PASylation technology (Schlapschy et al. 2013) led to a considerable extension of the PK for PTE-2 and PTE-3. This effect was most pronounced for the single-chain heterodimeric enzyme PTE-2 which has its both subunits linked by a 100 residue PAS linker (Escher et al. 2020).

### Calculated VX hydrolysis

From our previous studies, it was known that PTE-2 and PTE-3 show high catalytic efficiencies (*k*_cat_/*K*_M_) towards VX hydrolysis in vitro (Table 2), which are by three orders higher than the one of wild-type PTE (PTE-1), whose *k*_cat_/*K*_M_ value of 0.02 × 10^6^ M^−1^ min^−1^ for VX was determined in the present study. The maximum PTE plasma concentrations (*C*_max_), which were obtained from the PK analysis, allowed us to estimate half-lives of VX hydrolysis in vivo $\left( {{\text{VX~}}t_{{{1 \mathord{\left/ {\vphantom {1 2}} \right. \kern-\nulldelimiterspace} 2}}} } \right)$ which were below 10 s for the engineered enzymes but above 1 h for wild-type PTE (Table 2).

**Table 2:** Calculated VX degradation by PTE variants

Group	*k*_cat_/*K*_M_ (M^−1^ min^−1^)	*C*_max_ (mol L^−1^)	*t*_1/2_ (s)	Time to 96% VX degradation (s)
PTE-1	0.02 × 10^6^	5.7 × 10^–7^	3632	18,200
PTE-2.1	21 × 10^6^	2.4 × 10^–7^	8.2	41.1
PTE-2.2	21 × 10^6^	6.3 × 10^–7^	3.1	15.7
PTE-3	38 × 10^6^	4.6 × 10^–7^	2.4	11.8

*C*_max_ was taken from the PTE plasma profile of the therapeutic efficacy study. Degradation half-life was calculated with Eq. (1) and time to 96% VX degradation was estimated as 5 ×* t*_1/2_

### Therapeutic efficacy study

#### Clinical signs and survival

Subcutaneous injection of 25 µg kg^−1^ (~ 2LD_50_) VX (Misik et al. 2015; Myhrer et al. 2015) resulted in signs of chewing and an early onset of local muscle fasciculations directly at the injection site in all animals of the VX control, PTE-1 and PTE-2.1 groups. Chewing was observed in one single animal each of the PTE-2.2 and PTE-3 groups and local fasciculations were seen directly at the VX injection site in two animals of the PTE-2.2 group and in five animals of the PTE-3 group (data not shown).

**Fig. 3:**
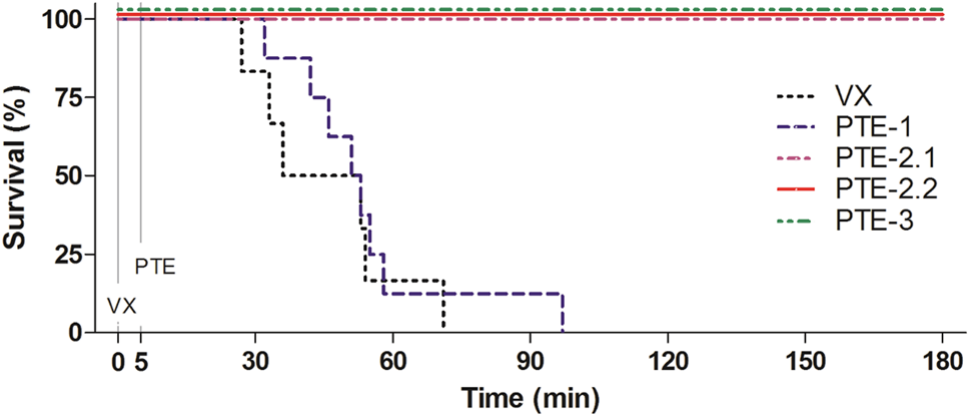
Survival plot showing the survival of rats after VX exposure (25 µg kg^−1^ s.c.) without treatment, for the VX control group, and with PTE treatment (i.v.), 5 min after intoxication, for the groups PTE-1 (1.0 mg kg^−1^ i.v.), PTE-2.1 (1.3 mg kg^−1^ i.v.), PTE-2.2 (2.6 mg kg^−1^ i.v.) and PTE-3 (1.4 mg kg^−1^ i.v.)

**Fig. 4:**
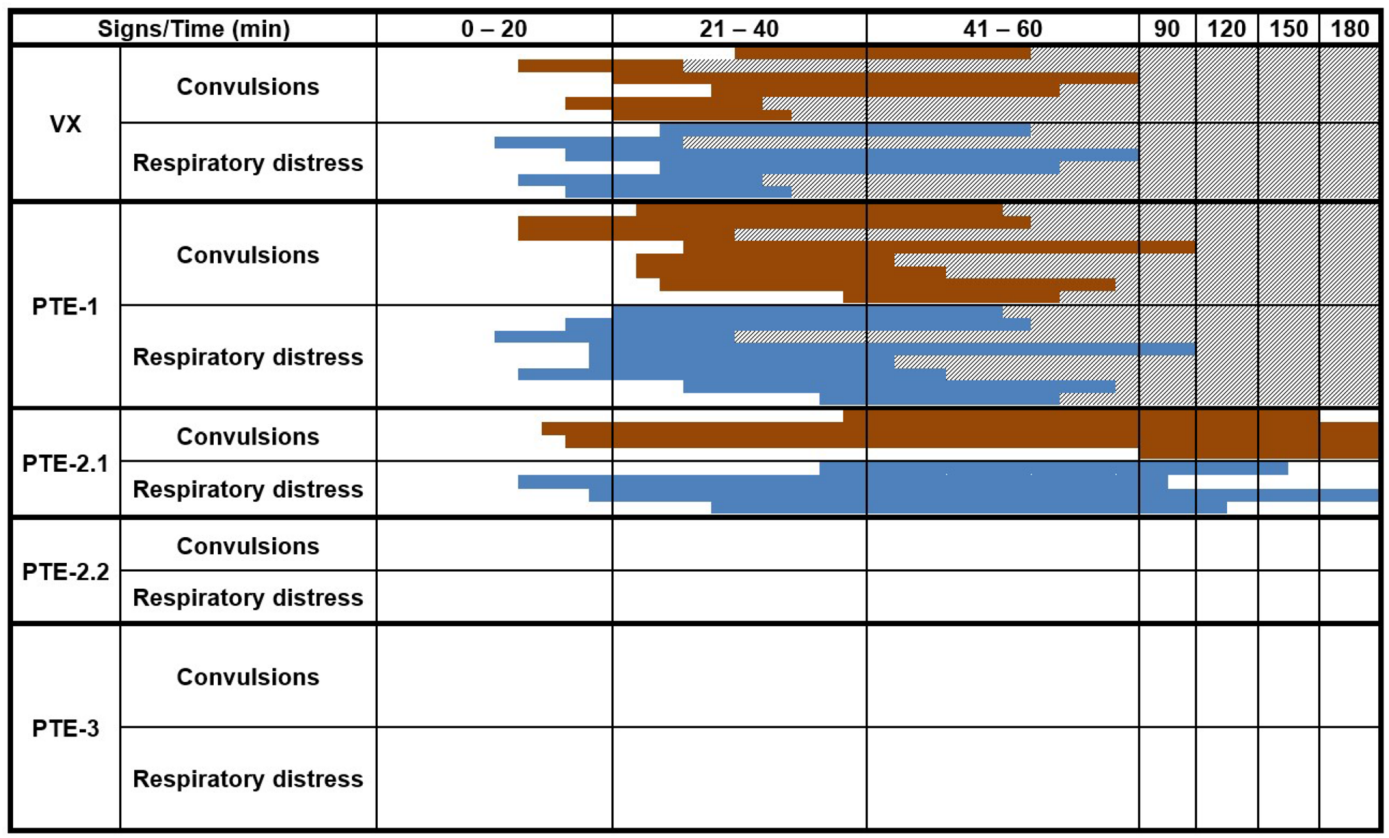
Clinical signs and survival of VX exposed rats without treatment and with post-exposure treatment of PTE-1, PTE-2 and PTE-3. Time of survival is given in minutes and each line represents an individual animal. Time of death is shown by grey, hatched lines. The signs are subdivided into convulsions (local, generalized) and respiratory distress (labored breathing, respiratory depression). 25 µg kg^−1^ VX s.c. (VX); 25 µg kg^−1^ VX s.c. followed after 5 min by 1.0 mg kg^−1^ PTE-1 i.v. (PTE-1); 25 µg/kg VX s.c. followed after 5 min by 1.3 mg kg^−1^ PTE-2 i.v. (PTE-2.1); 25 µg kg^−1^ VX s.c. followed after 5 min by 2.6 mg kg^−1^ PTE-2 i.v. (PTE-2.2); 25 µg kg^−1^ VX s.c. followed after 5 min by 1.4 mg kg^−1^ PTE-3 i.v. (PTE-3)

All degrees of convulsions, from uncontrolled muscle movements of head to forelimbs, hind legs and the entire body, were noted. Furthermore, respiratory distress was observed including the whole spectrum from labored breathing to respiratory depression. Over time, all animals of the VX control group and of the PTE-1 therapy group developed increasingly severe signs of a cholinergic crisis which persisted until death (Fig. 4). All animals of the VX control group died between 27 and 71 min after subcutaneous VX injection (mean: 46 ± 17 min). The animals of the PTE-1 therapy group survived only slightly longer, between 32 and 97 min (mean: 54 ± 19 min) after s.c. VX injection (Fig. 3). In contrast, all rats treated with PTE-2.1, PTE-2.2 and PTE-3 survived until the end of the observation period of 180 min (Fig. 3). Only one of four VX poisoned rats treated with PTE-2.1 showed a longer-lasting dyspnea until the end of the observation period, whereas convulsions persisted in three of four rats (Fig. 4). Notably, none of the animals treated with PTE-2.2 and PTE-3 showed convulsions or respiratory distress as severe clinical signs throughout the experiment (Fig. 4).

#### Erythrocyte AChE activity

**Fig. 5:**
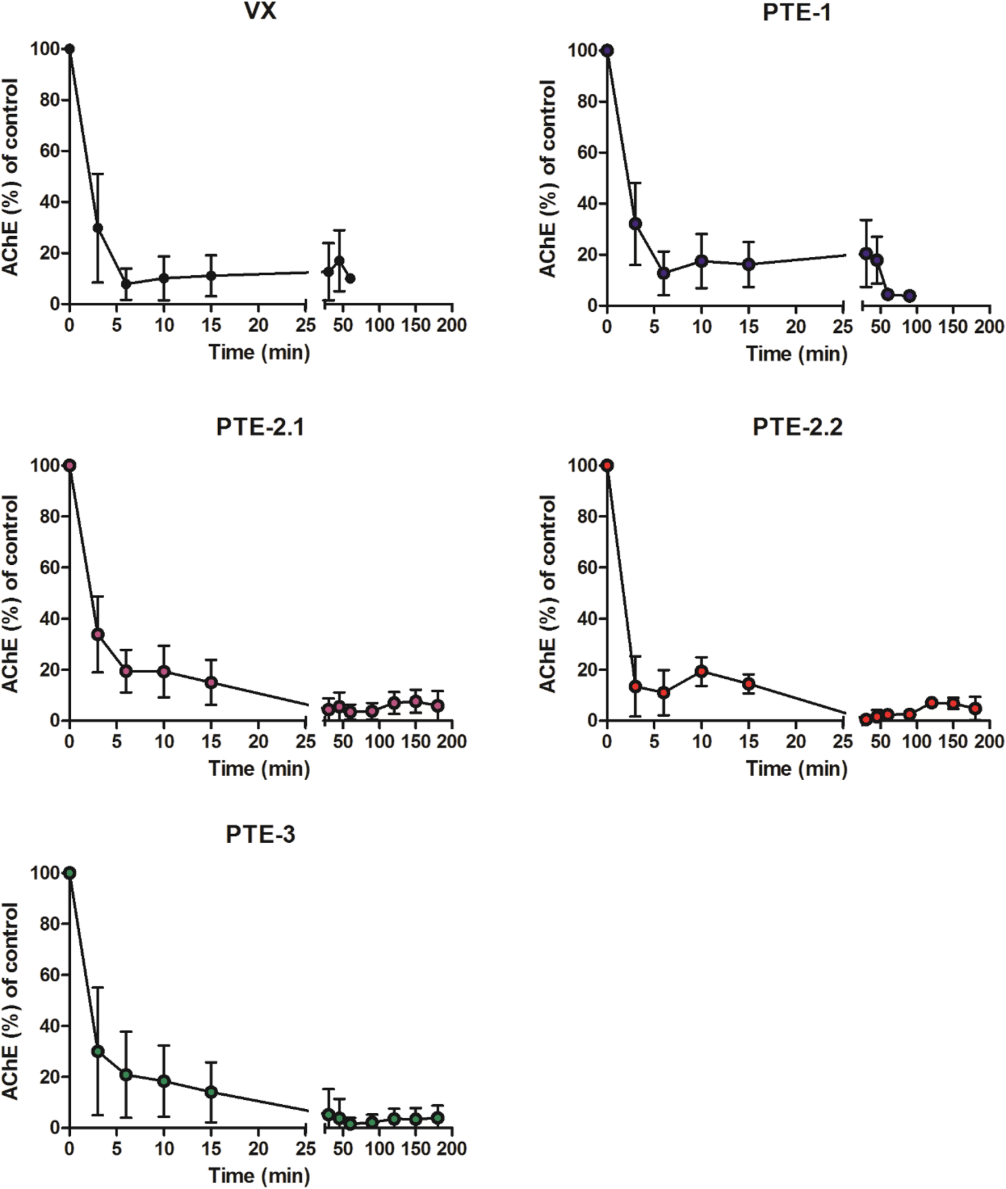
Time-dependent changes of erythrocyte AChE activity. 25 µg kg^−1^ VX s.c. (VX); 25 µg kg^−1^ VX s.c. followed after 5 min by 1.0 mg kg^−1^ PTE-1 i.v. (PTE-1); 25 µg kg^−1^ VX s.c. followed after 5 min by 1.3 mg kg^−1^ PTE-2 i.v. (PTE-2.1); 25 µg kg^−1^ VX s.c. followed after 5 min by 2.6 mg kg^−1^ PTE-2 i.v. (PTE-2.2); 25 µg kg^−1^ VX s.c. followed after 5 min by 1.4 mg kg^−1^ PTE-3 i.v. (PTE-3). Data are shown as % of solvent control activity (mean ± SD)

Erythrocyte AChE activities of animals prior to VX exposure and PTE treatment were 147 ± 14 mU µmol^−1^ Hb (PTE-1 treatment group), 141 ± 15 mU µmol^−1^ Hb (PTE-2.1 treatment group), 148 ± 21 mU µmol^−1^ Hb (PTE-2.2 treatment group), and 148 ± 16 mU µmol^−1^ Hb (PTE-3 treatment group). VX poisoning resulted in a rapid decrease of erythrocyte AChE activity in all groups and could not be prevented by administration of either PTE mutant (Fig. 5).

#### Tissue AChE activity

**Fig. 6:**
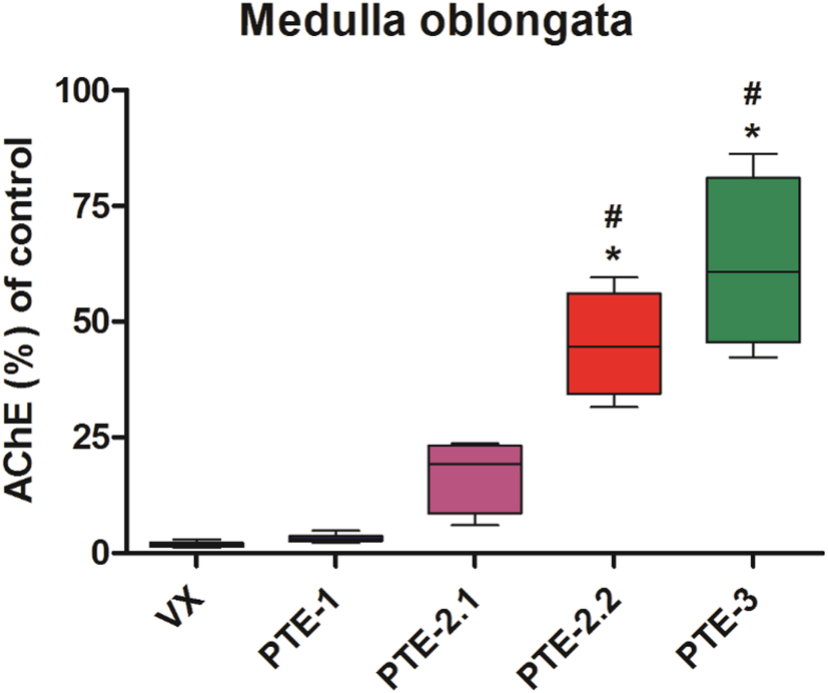
Medulla oblongata AChE activity of the VX control group and the PTE-1, PTE-2, PTE-2.2 and PTE-3 treated rat groups. Data are presented as % of pre-exposure control AChE activity as mean values ± SD using box plots with min to max whiskers. (*p < 0.05 versus VX control group; ^#^*p* < 0.05 versus PTE-1 group)

**Fig. 7:**
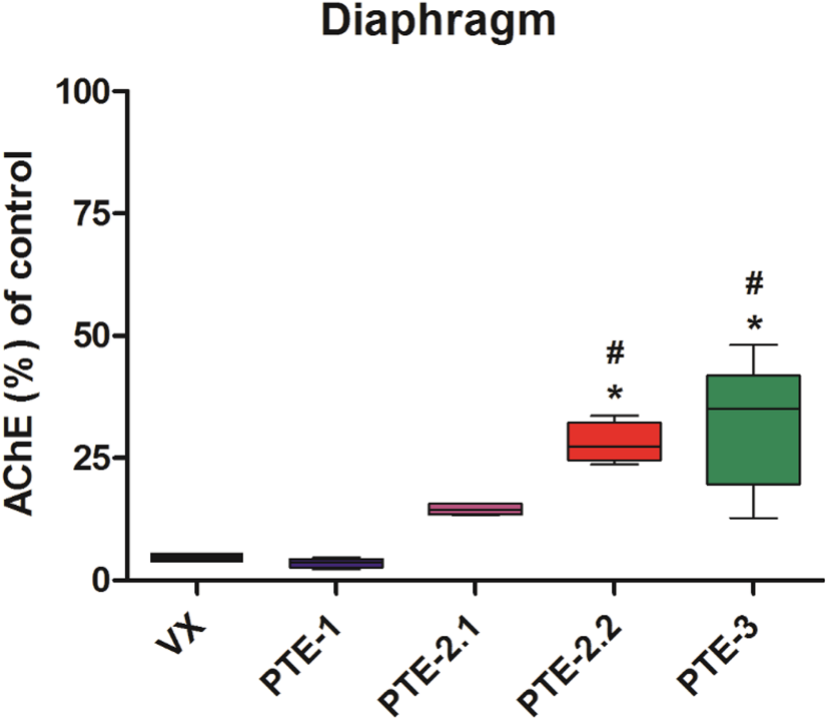
Diaphragm AChE activity of the VX control group and the PTE-1, PTE-2, PTE-2.2 and PTE-3 treated rat groups. Data are presented as % of pre-exposure control AChE activity as mean values ± SD using box plots with min to max whiskers. (*p < 0.05 versus VX control group; ^#^*p* < 0.05 versus PTE-1 group)

VX poisoning resulted in an almost complete inhibition of medulla oblongata and diaphragm AChE activity (Figs. 6, 7). Therapy with PTE-1 had no protective effect whereas administration of PTE-2 and PTE-3 resulted in a partial preservation of AChE activity in these tissues. This effect was statistically significant in the PTE-2.2 and PTE-3 groups compared to VX and PTE-1 (Figs. 6, 7).

#### VX blood concentration

**Fig. 8:**
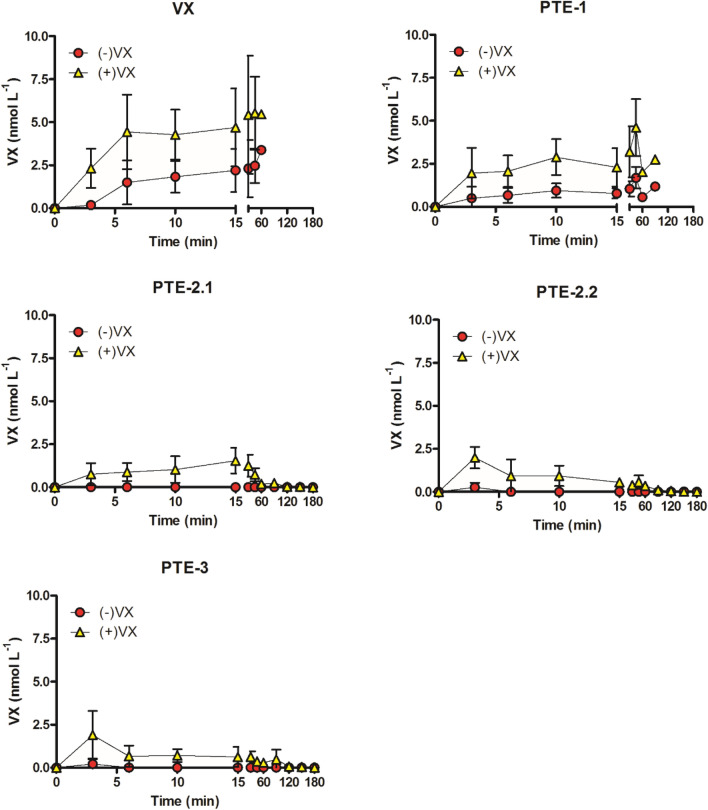
Concentration of VX enantiomers in whole blood samples. 25 µg kg^−1^ VX s.c. (VX); 25 µg kg^−1^ VX s.c. followed after 5 min by 1.0 mg kg^−1^ PTE-1 i.v. (PTE-1); 25 µg kg^−1^ VX s.c. followed after 5 min by 1.3 mg kg^−1^ PTE-2 i.v. (PTE-2.1); 25 µg kg^−1^ VX s.c. followed after 5 min by 2.6 mg kg^−1^ PTE-2 i.v. (PTE-2.2); 25 µg kg^−1^ VX s.c. followed after 5 min by 1.4 mg kg^−1^ PTE-3 i.v. (PTE-3). Data are given in nmol L^−1^ per VX enantiomer as mean values ± SD

The quantification of VX enantiomers in whole blood samples revealed a rapid increase of (+)-VX and a delayed increase of the more toxic (−)-VX enantiomer (Fig. 8). In the VX control and PTE-1 treatment groups the concentration of both enantiomers increased during the further course of the experiment (Fig. 8). The analysis of (+)-VX in the VX control group showed a sharp increase in concentration up to 4.4 nmol L^−1^ within the first 6 min after VX administration and a maximum concentration of 5.4 nmol L^−1^. The (−)-VX concentration showed a delayed increase with 1.5 nmol L^−1^ at the sampling time (6 min) and a lower maximal concentration of 3.4 nmol L^−1^ (Fig. 8). In contrast, administration of PTE-2 and PTE-3 5 min after VX challenge resulted in an ongoing hydrolysis, resulting in lower concentrations of (−)-VX (and (+)-VX) (Fig. 8). PTE-2.2 and PTE-3 treatment groups showed (+)-VX peak concentrations of 2.0 nmol L^−1^ and 1.9 nmol L^−1^, respectively, at 3 min with a subsequent decrease in concentration. The (−)-VX concentration exhibited only a very slight increase up to a maximum of 0.3 nmol L^−1^ for the PTE-2.2 treatment group and 0.2 nmol L^−1^ for the PTE-3 treatment group at 3 min, with subsequent concentration below the quantification limit (Fig. 8).

## Discussion

This study demonstrates the efficacy of post-exposure therapy of VX poisoned animals with the new PTE mutants PTE-2 and PTE-3. PTE-3 is a hyperactive stabilized homodimeric enzyme (Job et al. 2020) carrying two PAS(200) tags (Schlapschy et al. 2013) whereas PTE-2 is a single-chain version with two distinct engineered active sites (Escher et al. 2020) whose two subunits are linked by a PAS(100) spacer and which is equipped with one PAS(200) tag (Table 1).

Efficient OP detoxification requires a sufficiently high catalytic activity, which can be determined in vitro (*k*_cat_/*K*_m_). Previous studies have postulated that a *k*_cat_/*K*_m_ of at least 50 × 10^6^ M^−1^ min^−1^ is needed for successful therapy of VX poisoning at a reasonable enzyme dose of 1 mg kg^−1^ (Ashani et al. 2016; Worek et al. 2016a). Lower degradation rates or lower enzyme doses may still ensure survival but not prevent severe toxic effects.

Knowledge of the *k*_cat_/*K*_m_ values and the PTE plasma concentration enables the calculation of VX degradation half-lives (Table 2) (Worek et al. 2014). Our results demonstrate that the recombinant wild-type PTE from *Brevundimonas diminuta* (PTE-1), with an in vitro* k*_cat_/*K*_m_ value of 0.02 × 10^6^ M^−1^ min^−1^, was ineffective in vivo to treat VX poisoning. Throughout the experiment the PTE-1 treatment group revealed increasingly severe toxic signs and circulatory depression (data not shown) similar to the VX control group (Fig. 4). Our findings for PTE-1 are in line with prior data from Masson and Rochu (2009) and Kolakowski et al. (1997), who determined a negligible protective effect of wild-type PTE against V-type nerve agents.

In contrast, the novel engineered enzyme PTE-3, with an in vitro* k*_cat_/*K*_m_ of 38.1 × 10^6^ M^−1^ min^−1^, was able to prevent both mortality and severe toxicity in vivo. In comparison, the bispecific variant PTE-2, with an in vitro* k*_cat_/*K*_m_ of 20.7 × 10^6^ M^−1^ min^−1^, was able to prevent mortality but not severe toxicity (convulsions, respiratory distress) at the initial dose of 1 mg kg^−1^ (Fig. 4). However, administration of a doubled PTE-2 dose was successful with regard to preventing severe toxic signs and ensuring survival.

The therapeutic success of the PTE-2 and PTE-3 enzymes is mainly based on their high catalytic activities. Available *k*_cat_/*K*_m_ values and PTE plasma concentrations allow a first approximation of the degradation half-time (Table 2). These data indicate a negligible VX degradation in a relevant time window by PTE-1, whereas PTE-2 (PTE-2.2) and PTE-3 show extremely short *t*_1/2_ values of 3.1 and 2.4 s, respectively. For a more detailed analysis, the extended circulation of the PASylated enzymes has to be taken into account, which further boosts the OP degradation as will be discussed below.

These theoretical considerations are supported by analyses of the VX enantiomer concentrations in vivo (Fig. 8). There was a gradual increase and persistence of both (−)- and (+)-VX in the VX control and PTE-1 groups, whereas in the PTE-2 and PTE-3 treatment groups (−)-VX levels were at or below the limit of quantification shortly after administration of PTE (Fig. 8). Notably, the (+)-VX level also decreased after PTE administration in these groups, which corresponds to previous in vitro data on the stereoselective degradation of VX by PTE-2 (BdPTE-7) and PTE-3 (BdPTE-4), showing a preferential but not exclusive hydrolysis of (−)-VX (Köhler et al. 2021).

The s.c. VX injection resulted in a delayed appearance of (−)-VX in the blood, relevant (−)-VX concentrations were first observed in the 6 min samples of the VX control and PTE-1 groups. This is in accordance with the slightly delayed inhibition of erythrocyte AChE activity and the emerging first signs of VX poisoning in the VX control group between 11 to 26 min (Fig. 4).

The difference in catalytic efficiency and therapeutic efficacy between the PTE variants is also reflected by AChE activities in the diaphragm and medulla oblongata (Figs. 6, 7). While a virtually complete AChE inhibition was observed in the VX control and PTE-1 groups, enzyme activity was partially preserved in the PTE-2 and PTE-3 groups. This indicates that distribution of VX from the central blood compartment into target tissues could not be fully prevented but was reduced substantially depending on the catalytic activity of the different PTE variants and/or concentrations, i.e. PTE-3 > PTE-2.2 > PTE-2.1 >> PTE-1.

Efficient detoxification requires not only a high catalytic activity of PTE but also a long biological half-life. This is of special importance in the case of VX and related agents since V-agents exhibit a high biological stability and long persistence in vivo (Goldsmith et al. 2017; Reiter et al. 2011, 2015; van der Schans et al. 2003). In fact, in the case of VX poisoning current standard therapy with atropine and oxime must be administered over a prolonged period of time to preserve a sufficient level of active AChE and to ensure survival (Joosen et al. 2010). Hence, extension of the residence time of PTE in blood is the second key for a successful treatment. The PTE variants PTE-2 and PTE-3 were modified using PASylation technology (Binder and Skerra, 2017) in different ways, as explained above (Table 1). Plasma half-life of the unmodified wild-type PTE-1 was only 39 min whereas PTE-2 and PTE-3 showed a half-life of 165 min and 94 min (Fig. 2), respectively, which demonstrates that the PAS-tag has the ability to extend plasma half-life considerably. In fact, even more prolonged circulation can be expected when using longer PAS polypeptides, e.g. with 600 residues (Schlapschy et al. 2013; Binder and Skerra 2017; Gebauer and Skerra 2018).

The present study was based on anesthetized rats and s.c. administration of VX, which was due to animal welfare considerations and refinement at this stage of in vivo investigation. Further studies in conscious animals with percutaneous VX exposure are needed to verify the results and to more precisely evaluate required PTE doses and, potentially, the need for repeated PTE administration. Such studies are also needed to define the window of opportunity, i.e. the maximum time between agent exposure and PTE administration without risking severe signs of toxicity.

In conclusion, this study has demonstrated the potential of the engineered phosphotriesterases PTE-2 and PTE-3 as post-VX exposure treatment in order to prevent systemic toxicity and mortality. PASylation of PTE variants led to extended plasma half-life of these enzymes which is of special importance in the case of persistent nerve agents. Hence, the engineered PTE mutants PTE-2 and PTE-3 offer promising candidates as catalytic bioscavengers and deserve further investigation in vivo.
